# Corrigendum

**DOI:** 10.1093/ehjdh/ztac048

**Published:** 2022-10-06

In the originally published version of the below-listed manuscripts the data availability statement was accidentally omitted. Data availability statements have now been added to the following papers.

- https://doi.org/10.1093/ehjdh/ztaa012
- https://doi.org/10.1093/ehjdh/ztaa018
- https://doi.org/10.1093/ehjdh/ztab009
- https://doi.org/10.1093/ehjdh/ztab011
- https://doi.org/10.1093/ehjdh/ztab032
- https://doi.org/10.1093/ehjdh/ztab034
- https://doi.org/10.1093/ehjdh/ztab038
- https://doi.org/10.1093/ehjdh/ztab051
- https://doi.org/10.1093/ehjdh/ztab082
- https://doi.org/10.1093/ehjdh/ztab108

